# Predictive importance of the visceral adiposity index and atherogenic index of plasma of all-cause and cardiovascular disease mortality in middle-aged and elderly Lithuanian population

**DOI:** 10.3389/fpubh.2023.1150563

**Published:** 2023-03-13

**Authors:** Abdonas Tamosiunas, Dalia Luksiene, Daina Kranciukaite-Butylkiniene, Ricardas Radisauskas, Diana Sopagiene, Martin Bobak

**Affiliations:** ^1^Institute of Cardiology, Medical Academy, Lithuanian University of Health Sciences, Kaunas, Lithuania; ^2^Department of Environmental and Occupational Medicine, Medical Academy, Lithuanian University of Health Sciences, Kaunas, Lithuania; ^3^Department of Family Medicine, Medical Academy, Lithuanian University of Health Sciences, Kaunas, Lithuania; ^4^Department of Radiology, Medical Academy, Lithuanian University of Health Sciences, Kaunas, Lithuania; ^5^Institute of Epidemiology and Health Care, University College London, London, United Kingdom

**Keywords:** visceral adiposity index, atherogenic index of plasma, cardiovascular diseases, mortality, gender

## Abstract

**Background:**

Two indices: visceral adiposity index (VAI) and atherogenic index of plasma (AIP) during several recent years were implemented into epidemiological studies for predicting of cardiovascular diseases (CVD) and mortality risk. Our study aimed to evaluate the association of VAI and AIP with the risk of all-cause and CVD mortality among the Lithuanian urban population aged 45–72 years.

**Methods:**

In the baseline survey (2006–2008), 7,115 men and women 45–72 years of age were examined within the framework of the international study Health, Alcohol and Psychosocial Factors in Eastern Europe (HAPIEE). Six thousand six hundred and seventy-one participants (3,663 women and 3,008 men) were available for statistical analysis (after excluding 429 respondents with the missed information on study variables) and for them, VAI and AIP were calculated. The questionnaire evaluated lifestyle behaviors, including smoking and physical activity. All participants in the baseline survey were followed up for all-cause and CVD mortality events until December 31st, 2020. Multivariable Cox regression models were applied for statistical data analysis.

**Results:**

After accounting for several potential confounders, higher levels of VAI (compared 5th quintile to 1st quintile) were associated with significantly higher CVD mortality in men [Hazards ratio (HR) = 1.38] and all-cause mortality in women (HR = 1.54) after 10-year follow-up. CVD mortality significantly increased in men with 0 the highest AIP quintile compared with that for the lowest quintile (HR = 1.40). In women, all-cause mortality was significantly higher for the 4th quintile of AIP as compared with the 1st quintile (HR = 1.36).

**Conclusions:**

High-risk VAI levels were statistically significantly associated with all-cause mortality risk in men and women groups. The higher AIP level (5th quintile vs. 1st quintile—in men and 4th quintile vs. 1st quintile—in women) was significantly associated with increased mortality from CVD in the men group and increased all-cause mortality in the women group.

## 1. Background

Cardiovascular diseases (CVD) are the leading cause of death in most countries of Europe and other regions of the World (1, 2). The mortality from diseases of the circulatory system in a population aged 0–64 years in Lithuania during the period 2001–2016 has been decreasing from 131 to 103 cases per 100,000 population (1). Both increasing and decreasing trends of CVD morbidity and mortality indicators in the population are closely related to changes in the prevalence of biological and lifestyle risk factors such as arterial hypertension, smoking, hyperlipidaemia, physical inactivity, overweight and obesity, and other factors (3–6). Obesity is well-known and highly prevalent in most regions of Europe risk factor for CVD (7). In Lithuania, the prevalence of obesity during the period from 1999 to 2009 increased by 4.2% in men aged over 20 years (from 26.1 to 27.2%) and by 1.9% in women (from 26.1 to 26.3%). In 2030, the obesity prevalence in Lithuania maybe 35.7% in men and 36.0% in women (7). Body mass index (BMI) is the most widely used measure of obesity both in epidemiological studies of chronic non-communicable diseases and in clinical practice. However, BMI similar to waist circumference (WC), and another simple anthropometric measurement, cannot measure visceral and subcutaneous fat levels. It is demonstrated that visceral adipose and subcutaneous adipose tissues are related to the risk of CVD (8). The level of visceral adipose tissue and subcutaneous abdominal fat could be very precisely evaluated using magnetic resonance imaging (MRI) or computed tomography (CT), but these methods are very expensive to be applied in epidemiological studies of CVD or even in the practice of family doctors (9). Amato et al. a decade ago identified the visceral adiposity index (VAI) (10) which is calculated using a mathematical model and evaluates the level of visceral adipose tissue. The model uses both anthropometric (BMI and WC) and lipid parameters [triglyceride and high-density lipoprotein (HDL) cholesterol concentrations]. This index is also gender-specific: calculated separately for men and women using in the model different coefficients.

The atherogenic index of plasma (AIP) is another index quite recently been implemented in some epidemiologic studies of CVD (11–13). AIP is a logarithmic conversion of triglycerides into HDL cholesterol ratio, which as results of some epidemiological studies show is a stronger predictor of CVD risk as compared to individual lipid risk factors [total cholesterol, triglycerides, HDL cholesterol, and low-density lipoprotein (LDL) cholesterol] (14–16).

The association between individual lipid risk factors, overweight, and obesity same as some individual anthropometric measurements with risk of CVD incidence and mortality in the Lithuanian population was quite intensively studied, analyzed, and presented (17–19). But to the best of our knowledge, no study assessing the association between VAI and AIP with risk of all-cause and CVD mortality not only in the Lithuanian population but also in populations in other Baltic Sea countries: Latvia and Estonia. Therefore, our cohort study aimed to evaluate the association of VAI and AIP with the risk of all-cause and CVD mortality among the Lithuanian urban population aged 45–72 years.

## 2. Materials and methods

### 2.1. Study sample

This prospective cohort study was performed as part of the international project Health, Alcohol and Psychosocial Factors in Eastern Europe (HAPIEE) (20). The baseline survey was carried out (during 2006–2008) on Kaunas city (Lithuania) men and women aged 45–72 years. The study sample of 10,980 individuals, stratified by gender and age group, was randomly selected from the National population register. Seven thousand one hundred and fifteen individuals responded to the invitation to participate in the baseline survey (the response rate was 65%). All participants in the baseline survey follow-up for all-cause and CVD mortality events until December 31st, 2020. A total of 6,671 participants (3,663 women and 3,008 men) were available for statistical analysis after excluding 429 respondents with the missed information on study variables. Exclusion criteria: respondents for whom the nurses could not take a blood sample; respondents who refused to give blood for tests, and respondents who did not fill in the questionnaires correctly. The study was approved by the Kaunas Regional Biomedical Research Ethics Committee, Lithuania (11 January 2005; No. 05/09) and by the Ethics Committee at University College London, UK. Written informed consent was obtained from all study participants.

### 2.2. Variables determined using a standard questionnaire

Sociodemographic factors (age and education), lifestyle factors (smoking habits and physical activity), angina pectoris, and the history of CVD [previous coronary heart disease (CHD) and stroke] were determined at the baseline survey using a standard questionnaire. The reliability and validity of the questionnaire were checked during a pilot study.

The education of the participants was categorized into 2 groups: (1) secondary, vocational, or lower education; (2) college and university education.

Smoking habits were categorized as never smoking, former smoking, and current regular smoking (regular smoking at least 1 cigarette per day).

The physical activity of the participants in their leisure time was assessed using 5 questions in the standard questionnaire. Physical activity was calculated by summarizing time spent per week during leisure time separately in autumn-winter and spring-summer seasons for activities such as walking, gardening, maintenance of the house, and other physical activities. The participants were divided into three equal groups (tertiles) according to their mean length of time spent per week on physical activities. The first tertile maximal cut-off was 10 h per week. This cut-off was used for determining of insufficient physical activity of study participants.

To assess the history of previous myocardial infarction of the participants, 2 questions from the standard questionnaire were asked: “Has a doctor ever told you that you have had a myocardial infarction?” and “Has a doctor ever told you that you have had a stroke?”. Angina pectoris was evaluated by G. Rose's questionnaire (21).

### 2.3. Anthropometric measurements

Height, weight, and WC were measured directly by trained nurses. Weight was measured, with participants minimally clothed without shoes, using medical scales. Weight values were recorded to the nearest 100 g. The height of participants (without shoes) was measured with an accuracy of one centimeter, using a stadiometer. WC was measured at the midpoint between the lower rib and the iliac crest over light clothing, using a tape meter. Measurements of the WC were recorded to the nearest 0.5 cm. BMI (kg/m^2^) was calculated as weight (kg) divided by the square of the height (m^2^).

### 2.4. Other clinical and laboratory measurements

Blood pressure (BP) was measured three times with an oscillometric device (Omron M5-1) after at least 5 min of rest in a seated position, and mean values of systolic BP and diastolic BP were taken.

A resting electrocardiogram (ECG) was recorded in the 12 standard leads, with calibration of 10 mm per 1 mV and a paper speed of 25 mm per s. ECG records were read by 2 independent experienced coders (trained cardiologists) using the 1982 edition of the Minnesota Code (MC) (22).

Blood samples were drawn for the measurement of total cholesterol, HDL cholesterol, LDL cholesterol, and triglyceride levels the morning after study participants fasted overnight. All these biochemical determinations were performed in the same laboratory (the WHO Regional Lipid Reference Center, Institute of Clinical and Experimental Medicine, Prague (Czech Republic) using standard laboratory methods. The concentration of glucose in capillary blood was determined by a Glucotrend glucometer (23).

VAI score was calculated according to the definition of Amato et al. (10) using the following sex-specific equations where triglycerides and HDL-cholesterol levels are expressed in mmol/L:

Males: VAI = [WC (cm)/(39.68 + 1.88 × BMI (kg/m^2^))] × (triglycerides/1.03) × (1.31/HDL cholesterol)
Females: VAI = [WC (cm)/(36.58 + 1.89 × BMI (kg/m^2^))])] × (triglycerides/0.81) × (1.52/HDL cholesterol)
The AIP was calculated using the formula proposed by Frohlich and Dobiasova (11) (log_10_ (triglycerides (mmol/L)/HDL cholesterol (mmol/L))).

### 2.5. Definitions

Arterial hypertension was defined as systolic BP ≥ 140 mmHg and/or diastolic BP ≥ 90 mmHg, or usage of anti-hypertensive medication during last 2 weeks (24).

Increased level of total serum cholesterol was determined as total cholesterol concentration ≥5.0 mmol/L and increased fasting glucose level as glucose concentration in capillary blood ≥6.1 mmol/L (25, 26).

Insufficient physical activity was determined in the case when the mean time spent per week by study participants during leisure time for physical activities was lower than 10 h.

The participants were ranked from the lowest to the highest values of VAI and AIP and divided into five equal groups (quintiles) according to the levels of these variables (Table 1).

**Table 1:** Visceral adiposity index (VAI) and atherogenic index of plasma (AIP) levels.

	**Men**		**Women**	
	**Min–max**	**Mean (SD)**	**Min–max**	**Mean (SD)**
**VAI quintiles**				
1st quintile	0.16 to 0.69	0.51 (0.12)	0.27 to 0.85	0.65 (0.14)
2nd quintile	0.69 to 1.03	0.85 (0.10)	0.85 to 1.22	1.03 (0.11)
3rd quintile	1.03 to 1.44	1.22 (0.12)	1.22 to 1.71	1.44 (0.14)
4th quintile	1.44 to 2.21	1.78 (0.23)	1.71 to 2.57	2.08 (0.24)
5th quintile	2.21 to 19.3	3.68 (1.94)	2.57 to 24.0	4.13 (2.03)
**AIP quintiles**				
1st quintile	−0.87 to −0.27	−0.40 (0.11)	−0.76 to −0.30	−0.42 (0.10)
2nd quintile	−0.27 to −0.10	−0.18 (0.05)	−0.30 to −0.15	−0.23 (0.04)
3rd quintile	−0.10 to 0.04	−0.03 (0.04)	−0.15 to 0.01	−0.08 (0.04)
4th quintile	0.04 to 0.22	0.13 (0.05)	0.01 to 0.16	0.07 (0.05)
5th quintile	0.22 to 1.15	0.40 (0.16)	0.16 to 1.10	0.33 (0.15)

SD, standard deviation.

Coronary heart disease (CHD) at baseline was determined by: (1) a documented history of myocardial infarction (MI) and/or ischemic changes on ECG coded by MC 1–1 or 1–2 (22); (2) angina pectoris as defined by G. Rose's questionnaire (without MI and/or MC 1–1 or 1–2) (21); (3) ECG findings coded by MC 1–3, 4–1, 4–2, 4–3, 5–1, 5–2, 5–3, 6–1, 6–2, 7–1, or 8–3 (without MI and/or MC 1–1, 1–2 and without angina pectoris). The previous stroke was determined according to a documented history of stroke.

CVD included CHD and/or stroke which were determined at the baseline survey.

### 2.6. Mortality outcome

We used data from the Kaunas mortality register based on death certificates with follow-up through December 31, 2020. Cause of death was categorized using the International Classification of Diseases, 10th Edition (ICD-10). All causes of death included ICD-10 codes A00-Z99. CVD-specific mortality was categorized using codes I00-I99.

### 2.7. Statistical analysis

All statistical analysis was performed using IBM SPSS Statistics (Version 27.0) (IBM Corp. Released 2020. IBM SPSS Statistics for Windows, Version 27.0. Armonk, NY, USA). We performed an analysis of study data separately for men and women. All descriptive characteristics [proportions, means, and standard deviations (SD)] were calculated and presented across the groups by vital status at the end of follow-up in two ways (alive and died from all causes; alive, died from CVD and died from other causes). Differences between groups were detected by independent sample *t*-test and ANOVA analysis with Bonferroni corrections for continuous variables. A Chi-squared test and *Z*-test with Bonferroni corrections were used for determining differences in categorical variables. *P*-values < 0.05 were considered statistically significant.

We fit Cox proportional hazards regression models to estimate the hazard ratio (HR) and 95% confidence interval (CI) for quintiles of VAI and AIP with all-cause and CVD mortality. The participants who previously had CVD (CHD or/and stroke) were removed from the analysis of CVD mortality risk. Standardized multivariable Cox regression models were used to evaluate the effect size of VAI and AIP with three steps. Model 1 includes a single VAI and AIP quintile with 1st quintile as the reference group. In Model 2 age as, a continuous variable, is added. Model 3 was adjusted for all the variables in Model 2 plus education, physical activity, smoking status, and biological factors (arterial hypertension, total cholesterol, and fasting glucose) (all categorical). Risk of all-cause and CVD mortality was also assessed using the same 3 Cox regression models when VAI and AIP values in the model changed per 1 quintile.

## 3. Results

The mean duration and SD of the follow-up of the participants were 12.6 ± 2.79 years. During the follow-up, there were 1,444 all-cause deaths (882 men and 562 women) and 682 deaths from CVD [414 men and 268 women (232 and 150 deaths, respectively, among participants without CVD at baseline survey)] registered.

The characteristics of the respondents at the baseline survey, according to their survival status are presented in Tables 2, 3. Men and women who died from all-cause deaths and CVD deaths during the follow-up period were significantly older and less educated at the baseline survey than those alive at the end of the follow-up. During the initial study, the age-adjusted means of some biological factors, such as systolic and diastolic BP, triglycerides, the fasting glucose level had been higher, and HDL cholesterol level had been lower in men and women who died from all-cause deaths and CVD deaths during the follow-up period compared to those who were alive. Moreover, it was determined that the respondents who died from all-cause deaths and CVD deaths during the follow-up period had been more often diagnosed with diabetes mellitus, CVD, and arterial hypertension than those who were alive at the end of the follow-up. Men and women who died from all-cause deaths and CVD deaths during the follow-up period were lower physically active in their leisure time and their mean BMI and WC levels had been higher than those alive at the end of the follow-up. It's important, that mean levels of VAI, and AIP had been higher in men and women who died from all-cause deaths and CVD deaths during the follow-up period compared to those who were alive.

**Table 2:** Baseline characteristics by survival status of men of the Kaunas HAPIEE study (2006–2008).

Variables^*^	**Living status**		
	**Alive (*****n*** = **2,126)**	**All causes of death (*****n*** = **882)**	**Dead from CVD (*****n*** = **414)**
Age, years, mean (SD)	56.0 (7.46)	62.3 (7.55)^a^	63.1 (7.20)^a^
Systolic blood pressure, mmHg, mean (SD)	142.9 (20.0)	150.9 (23.4)^a^	153.7 (24.4)^a^
Diastolic blood pressure, mmHg, mean (SD)	92.3 (12.5)	94.8 (13.6)^a^	92.3 (12.5)^a^
Arterial hypertension, %	74.6	83.7^a^	86.3^a^
Total cholesterol, mmol/L, mean (SD)	5.81 (1.08)	5.71 (1.15)^a^	5.73 (1.13)
HDL cholesterol, mmol/L, mean (SD)	1.41 (0.37)	1.39 (0.39)	1.34 (0.38)^a^
Triglyceride, mmol/L, mean (SD)	1.48 (0.89)	1.55 (0.98)	1.69 (0.18)^a^
Fasting blood glucose, mmol/L, mean (SD)	5.72 (1.07)	6.01 (1.66)^a^	6.25 (2.00)^a^
Body mass index, kg/m^2^, mean (SD)	28.3 (4.30)	29.0 (5.19)^a^	29.9 (5.39)^a^
WC, cm, mean (SD)	94.3 (11.8)	97.9 (13.7)^a^	100.3 (13.8)^a^
VAI, mean (SD)	1.59 (1.37)	1.75 (1.71)^a^	2.00 (2.12)^a^
AIP, mean (SD)	−0.02 (0.29)	0.001 (0.30)	0.047 (0.32)^a^
Diabetes mellitus, %	5.3	14.4^a^	18.7^a^
CVD, %	12.6	33.9^a^	43.3^a^
Physical active in leisure time, %	67.5	61.3^a^	59.5^a^
**Smoking habits, %**			
Smokers, %	34.0	36.5	33.3
Never smokers + former smokers, %	66.0	63.5	66.7
**Education, %**			
Secondary + vocational	42.7	58.8^a^	58.3^a^
College + university	57.3	41.2^a^	41.7^a^

^*^All variables are age-adjusted.

^a^p < 0.05 compared to the alive group.

CVD, cardiovascular diseases; HAPIEE, Health, Alcohol and Psychosocial factors In Eastern Europe; HDL, high-density lipoprotein; WC, waist circumference; AI, visceral adiposity index; AIP, atherogenic index of plasma.

**Table 3:** Baseline characteristics by survival status of women of the Kaunas HAPIEE study (2006–2008).

Variables^*^	**Living status**		
	**Alive (*n* = 3,101)**	**All causes of death (*n* = 562)**	**Dead from CVD (*n* = 268)**
Age, years, mean (SD)	56.4 (7.53)	63.4 (7.48)^a^	66.5 (5.43)^a^
Systolic blood pressure, mmHg, mean (SD)	133.3 (19.9)	143.4 (24.3)^a^	148.9 (23.9)^a^
Diastolic blood pressure, mmHg, mean (SD)	87.3 (11.4)	90.9 (12.8)^a^	92.5 (12.9)^a^
Arterial hypertension, %	65.5	79.7^a^	88.3^a^
Total cholesterol, mmol/L, mean (SD)	6.06 (1.12)	5.96 (1.14)	6.03 (1.19)
HDL cholesterol, mmol/L, mean (SD)	1.60 (0.37)	1.50 (0.36)^a^	1.47 (0.34)^a^
Triglyceride, mmol/L, mean (SD)	1.39 (0.75)	1.53 (0.81)^a^	1.60 (0.94)^a^
Fasting blood glucose, mmol/L, mean (SD)	5.73 (0.99)	6.16 (1.77)^a^	6.34 (2.03)^a^
Body mass index, kg/m^2^, mean (SD)	29.4 (5.56)	31.4 (6.43)^a^	31.9 (6.82)^a^
WC, cm, mean (SD)	87.9 (13.4)	93.9 (15.1)^a^	95.1 (15.6)^a^
VAI, mean (SD)	1.74 (1.49)	2.08 (1.78)^a^	2.25 (2.18)^a^
AIP, mean (SD)	−0.098 (0.27)	−0.026 (0.26)^a^	−0.006 (0.27)^a^
Diabetes mellitus, %	6.1	17.5^a^	24.1^a^
CVD, %	17.7	33.6^a^	43.6^a^
Physical active in leisure time, %	81.7	74.0^a^	68.4^a^
**Smoking habits, %**			
Smokers	14.0	9.2^a^	5.3^a^
Never smokers + former smokers	86.0	90.8^a^	94.7^a^
**Education, %**			
Secondary + vocational	35.2	51.8^a^	59.9^a^
College + university	64.8	48.2^a^	40.1^a^

^*^All variables are age-adjusted.

^a^p < 0.05 compared to the alive group.

CVD, cardiovascular diseases; HAPIEE, Health, Alcohol and Psychosocial factors In Eastern Europe; HDL, high-density lipoprotein; WC, waist circumference; VAI, visceral adiposity index; AIP, atherogenic index of plasm.

In Table 4, we present the multiple cox regression assessments of VAI in the prediction of risk of all-cause and cardiovascular mortality according to gender over 10 years. Based on the crude model (Model 1) assessments, the men with higher VAI levels (5th quintile) had a 1.26-fold increased risk of all-cause mortality and 1.78-fold increased mortality from CVD risk compared with men with lower VAI levels (1st quintile). An increase per quintile in the VAI significantly increased the risk of all-cause mortality (by 7%) and the risk of mortality from CVD (by 14%) in the men group. After additional adjustment for age (Model 2) the same risk of all-cause mortality and mortality from CVD remained statistically significant in the men group. However, after adjustment for age, education, physical activity, smoking status, and biological factors (Model 3) a significant relationship was determined for all-cause mortality risk per quintile of VAI (by 5%), and for risk of mortality from CVD (by 38%) than compared the men with higher VAI level (5th quintile) to men with lower VAI level (1st quintile).

**Table 4:** Risk of all-cause and cardiovascular mortality for visceral adiposity index (VAI) levels status according to gender over 10 years [follow-up for endpoints mean period (SD) 12.6 (2.75) years].

	**All-cause deaths**			**CVD deaths** ^*^		
**VAI level/cox models**	**HR**	**95% CI**	***p***	**HR**	**95% CI**	***p***
**Men**	(*n* = 882)			(*n* = 232)		
**Model 1**						
1st quintile	1			1		
2nd quintile	0.87	0.70–1.08	0.201	1.04	0.74–1.47	0.803
3rd quintile	1.07	0.86–1.32	0.552	**1.47**	1.07–2.01	0.018
4th quintile	1.03	0.83–1.27	0.780	1.16	0.83–1.61	0.398
5th quintile	**1.26**	1.03–1.55	0.024	**1.78**	1.31–2.42	< 0.001
Per quintile	**1.07**	1.02–1.12	0.006	**1.14**	1.06–1.22	< 0.001
**Model 2**						
1st quintile	1			1		
2nd quintile	0.85	0.68–1.06	0.141	1.02	0.73–1.44	0.903
3rd quintile	0.99	0.81–1.23	0.990	1.37	0.995–1.88	0.054
4th quintile	1.01	0.82–1.25	0.935	1.13	0.81–1.58	0.473
5th quintile	**1.31**	1.07–1.60	0.010	**1.85**	1.36–2.52	< 0.001
Per quintile	**1.08**	1.03–1.13	0.002	**1.15**	1.07–1.24	< 0.001
**Model 3**						
1st quintile	1			1		
2nd quintile	0.91	0.73–1.14	0.411	1.05	0.75–1.48	0.788
3rd quintile	0.99	0.81–1.23	0.982	1.28	0.93–1.76	0.134
4th quintile	1.01	0.82–1.25	0.923	0.94	0.67–1.32	0.721
5th quintile	1.21	0.98–1.49	0.080	**1.38**	1.00–1.90	0.047
Per quintile	**1.05**	1.00–1.10	0.046	1.06	0.99–1.14	0.116
**Women**	(*n* = 562)			(*n* = 150)		
**Model 1**						
1st quintile	1			1		
2nd quintile	**1.74**	1.28–2.36	< 0.001	**1.93**	1.22–3.06	0.005
3rd quintile	**1.75**	1.29–2.37	< 0.001	**1.99**	1.26–3.15	0.003
4th quintile	**2.13**	1.59–2.87	< 0.001	**2.26**	1.44–3.54	< 0.001
5th quintile	**2.33**	1.74–3.12	< 0.001	**2.75**	1.78–4.25	< 0.001
Per quintile	**1.19**	1.12–1.26	< 0.001	**1.22**	1.12–1.33	< 0.001
**Model 2**						
1st quintile	1			1		
2nd quintile	**1.49**	1.09–2.02	0.011	1.55	0.98–2.45	0.060
3rd quintile	**1.41**	1.03–1.91	0.030	1.50	0.95–2.36	0.084
4th quintile	**1.60**	1.19–2.15	0.002	1.55	0.99–2.43	0.056
5th quintile	**1.72**	1.28–2.31	< 0.001	**1.87**	1.21–2.89	0.005
Per quintile	**1.11**	1.05–1.18	0.001	**1.12**	1.02–1.22	0.014
**Model 3**						
1st quintile	1			1		
2nd quintile	**1.48**	1.08–2.01	0.014	1.51	0.95–2.40	0.081
3rd quintile	1.35	0.99–1.84	0.057	1.28	0.81–2.04	0.291
4th quintile	**1.51**	1.11–2.04	0.008	1.30	0.83–2.06	0.256
5th quintile	**1.54**	1.13–2.08	0.006	1.50	0.96–2.36	0.078
Per quintile	**1.08**	1.01–1.15	0.020	1.06	0.962–1.16	0.254

^*^Among participants without CVD at baseline survey.

HR, hazard ratios; CI, confidence interval; CVD, cardiovascular diseases.

Model 1 unadjusted data. Model 2 adjusted for age. Model 3 adjusted for age, education, physical activity and smoking status, and biological factors (arterial hypertension, total cholesterol, and fasting glucose). For details see “Methods” and “Statistical analysis”.

Bold values means statistically significant difference of HR values compared with 1st quintile HR.

In the women group, an increase per quintile in the VAI significantly increased the risk of all-cause mortality (by 19%) and the risk of mortality from CVD (by 22%) (Model 1). Also, by increasing the quintile of VAI (2nd, 3rd, 4th, 5th quintile) the risk of all-cause mortality risk and CVD mortality risk increased compared with the lowest VAI quintile (1st quintile). However, after adjustment for age, education, physical activity, smoking status, and biological factors (Model 3) a significant relationship was determined only for all-cause mortality risk per quintile of VAI (by 8%) in the women group. Such a significant relationship was not determined for the risk of mortality from CVD in the women group.

In Table 5, we present the multiple Cox regression assessments of AIP in the prediction of risk of all-cause and CVD mortality according to gender over 10 years. Based on the crude model (Model 1) an increase per quintile in the AIP significantly increased the risk of all-cause mortality (by 5%) and the risk of mortality from CVD (by 13%) in the men group. After additional adjustment for age (Model 2) the same risk of all-cause mortality and mortality from CVD remained statistically significant in the men group. However, after adjustment for age, education, physical activity, smoking status, and biological factors (Model 3) the men with higher AIP levels (5th quintile) had a 1.40-fold increased risk of mortality from CVD risk compared with men with lower AIP level (1st quintile).

**Table 5:** Risk of all-cause and cardiovascular mortality for the atherogenic index of plasma (AIP) levels status according to gender over 10 years [follow-up for endpoints mean period (SD) 12.6 (2.75) years].

	**All-cause deaths**			**CVD deaths** ^*^		
**AIP level/cox models**	**HR**	**95% CI**	***p***	**HR**	**95% CI**	***p***
**Men**	(*n* = 882)			(*n* = 232)		
**Model 1**						
1st quintile	1			1		
2nd quintile	0.85	0.68–1.05	0.127	0.94	0.67–1.31	0.703
3rd quintile	0.98	0.79–1.21	0.830	1.36	0.99–1.85	0.055
4th quintile	0.96	0.78–1.19	0.709	1.00	0.72–1.40	0.995
5th quintile	1.19	0.97–1.46	0.092	**1.72**	1.27–2.32	< 0.001
Per quintile	**1.05**	1.00–1.10	0.039	**1.13**	1.06–1.21	< 0.001
**Model 2**						
1st quintile	1			1		
2nd quintile	0.82	0.66–1.01	0.064	0.90	0.64–1.26	0.539
3rd quintile	0.94	0.77–1.16	0.581	1.30	0.96–1.78	0.094
4th quintile	0.95	0.77–1.17	0.629	0.99	0.71–1.38	0.950
5th quintile	**1.24**	1.02–1.52	0.035	**1.81**	1.34–2.44	< 0.001
Per quintile	**1.06**	1.01–1.12	0.011	**1.15**	1.07–1.24	< 0.001
**Model 3**						
1st quintile	1			1		
2nd quintile	0.88	0.71–1.09	0.248	0.95	0.68–1.34	0.782
3rd quintile	0.95	0.77–1.18	0.655	1.25	0.91–1.71	0.171
4th quintile	0.96	0.78–1.19	0.698	0.85	0.61–1.20	0.354
5th quintile	1.16	0.95–1.43	0.152	**1.40**	1.03–1.91	0.033
Per quintile	1.04	0.99–1.09	0.102	1.07	0.99–1.15	0.079
**Women**	(*n* = 562)			(*n* = 150)		
**Model 1**						
1st quintile	1			1		
2nd quintile	**1.46**	1.09–1.97	0.012	**1.55**	1.00–2.39	0.047
3rd quintile	**1.54**	1.15–2.07	0.004	**1.63**	1.06–2.51	0.025
4th quintile	**1.88**	1.42–2.50	< 0.001	**1.85**	1.22–2.82	0.004
5th quintile	**1.92**	1.45–2.55	< 0.001	**2.08**	1.38–3.14	< 0.001
Per quintile	**1.16**	1.09–1.23	< 0.001	**1.17**	1.07–1.27	< 0.001
**Model 2**						
1st quintile	1			1		
2nd quintile	1.23	0.92–1.66	0.168	1.23	0.80–1.89	0.355
3rd quintile	1.31	0.98–1.76	0.070	1.33	0.87–2.05	0.189
4th quintile	**1.41**	1.06–1.87	0.019	1.27	0.83–1.93	0.273
5th quintile	**1.46**	1.10–1.93	0.009	1.47	0.97–2.21	0.069
Per quintile	**1.09**	1.02–1.15	0.007	1.08	0.99–1.18	0.094
**Model 3**						
1st quintile	1			1		
2nd quintile	1.25	0.92–1.68	0.150	1.19	0.77–1.84	0.433
3rd quintile	1.29	0.96–1.73	0.096	1.19	0.77–1.84	0.425
4th quintile	**1.36**	1.01–1.81	0.040	1.08	0.71–1.67	0.714
5th quintile	1.31	0.98–1.76	0.068	1.20	0.78–1.83	0.412
Per quintile	1.06	0.99–1.12	0.081	1.02	0.93–1.12	0.662

^*^Among participants without CVD at baseline survey.

HR, hazard ratios; CI, confidence interval; CVD, cardiovascular diseases.

**Model 1** unadjusted data. **Model 2** adjusted for age. **Model 3** adjusted for age, education, physical activity and smoking status, and biological factors (arterial hypertension, total cholesterol, and fasting glucose). For details see “Methods” and “Statistical analysis”.

Bold values means statistically significant difference of HR values compared with 1st quintile HR.

In the women group, an increase per quintile in the AIP significantly increased the risk of all-cause mortality (by 16%) and the risk of mortality from CVD (by 17%) (Model 1). Also, by increasing the quintile of AIP (2nd, 3rd, 4th, 5th quintile) the risk of all-cause mortality risk and CVD mortality risk increased compared with the lowest AIP quintile (1st quintile). However, after adjustment for age, education, physical activity, smoking status, and biological factors (Model 3) a significant association was not determined between AIP levels and all-cause mortality risk and the risk of mortality from CVD in the women group.

## 4. Discussion

In this study, we presented an independent association of two indices—VAI and AIP with risk of all-cause and CVD mortality in the middle-aged and elderly Lithuanian urban population.

We found that in Cox regression analyses, compared to the 1st VAI quintile, the 5th quintile was an independent positive predictor of all-cause and CVD mortality risk in males and females. This significant association remained after adjusting for several lifestyles and biological confounding factors for CVD mortality in men (HR = 1.38) and all-cause mortality in women (HR = 1.54). These data are consistent with previous studies in which researchers also found a positive association between increasing levels of VAI and risk of all-cause and CVD mortality (27, 28). Other researchers demonstrated results that higher VAI was related to the risk of the incidence of CVD and cancer (29, 30).

VAI indirectly indicates visceral adipose tissue (VAT) deposits and functions in the human body (10). This index could be used in epidemiological studies and even clinical practice instead of expensive diagnostic methods such as CT, MRI, or sonographic assessment (31, 32). The anthropometric measurements such as WC, waist-to-hip ratio or abdominal sagittal diameter also could be used for the evaluation of regional intraabdominal fat deposition, but those measurements are characterized by low accuracy (33). WC also could not differentiate VAT from subcutaneous adipose tissue in the abdomen which has a lower CVD risk level (29, 34). A similar indicator recently proposed for the evaluation of VAT is anthropometrically predicted VAT (34, 35). This indicator is also VAI sex-specific and the equations for calculation of the indicator include anthropometric measures (BMI, WC, and thigh circumference) and age. VAI is the more specific and precise index for evaluation of VAT as compared to anthropometrically predicted VAT because models for calculation of such includes not only anthropometric data (BMI and WC) but also lipid levels (HDL cholesterol and triglycerides) (10). Several studies demonstrated that VAI was a better predictor of the incidence and mortality risk as compared to BMI and WC in the prognostic models alone (28, 36). Whereas, some studies presented research data indicating that the impact of VAI was not significantly different for predicting CVD risk in women and type 2 diabetes in the non-diabetic population as compared with simply measured anthropometric biomarkers (30, 37). In this study, we did not compare the impact of VAI and such anthropometric measurements as BMI, WC, waist-to-hip ratio, or waist-to-height ratio on the CVD mortality risk. Data from our previous study showed that among participants from three surveys the anthropometric measurements (BMI, WC, waist-to-hip ratio, and waist-to-height ratio) changing per 1 SD in multivariable-adjusted Cox's regression model significantly increased CVD mortality risk in men (HR varied from 1.40 to 1.49) but not in women (18).

The AIP is a logarithmically converted ratio of triglycerides to HDL cholesterol (11). The results of our study showed that values of AIP were higher among males as compared to females. Such gender differences in AIP values were demonstrated in most similar studies (38, 39). The results of ten observational studies performed in China, Turkey, and South Korea demonstrated that higher AIP values may be independently associated with the odds of coronary artery disease (CAD) (40). The AIP could be also considered an independent predictor of CVD incidence and mortality risk (38, 41). Results from a large-scale nationwide population cohort study carried on in the Republic of Korea showed that AIP HRs for CVD risk were higher as compared to HRs of triglycerides and HDL cholesterol when those variables were applied in the regression model alone (42). Whereas, a study performed among 1,131 male patients with angiographically diagnosed CAD and without CAD found that the AIP predictive value for diagnosing CAD was not significantly different as compared with traditional blood lipids (43). Our results have shown a positive association between increasing values of AIP and risk of CVD mortality after adjustment to many confounders in men but not women responders. In women responders, the risk of all-cause mortality was significantly higher by 36% when the 4th quintile of AIP was compared with the 1st quintile. The association between AIP and CVD incidence and mortality is mainly explained by the correlation of the index with lipoprotein particle size: it is inversely related to LDL cholesterol particle diameter (44). Results of some studies showed that small dense LDL cholesterol particles were related to higher risk of CVD (45, 46). However, the methods for determining small dense LDL cholesterol particles are quite complicated and the cost-effectiveness of such methods is low, therefore those methods are not recommended for use in clinical practice and large-scale epidemiological studies of CVD (47).

VAI and API calculations are cheap methods and could be used not only in epidemiological studies but especially in clinical practice for cardiologists or endocrinologists instead of expensive diagnostic methods.

### 4.1. Strength and limitations of the study

The main strength of our study was: a large sample size, adjustment using many confounding variables, and a cohort study design. Our study also has some limitations. Despite many variables used in the adjustment procedure, it is still possible that some not measured confounding variables could interfere with a part of the associations between AIP or VAI and CVD or all-cause mortality risk. The findings of this study cannot be generalized to the Lithuanian countrywide population because our study was carried out only in an urban one-city a middle-aged and elderly population. Therefore, further epidemiological investigations are needed to be performed in different regions of the country. Finally, we used the baseline AIP and VAI levels for the assessment of the association of those indices with the risk of all-cause and CVD mortality. Any changes during the follow-up both of AIP, VAI, and confounding variables are missing. Despite the mentioned limitations, our study provided additional insight into the association between higher levels of these indices and the risk of mortality.

## 5. Conclusion

High-risk VAI levels were statistically significantly associated with all-cause mortality risk in men and women groups. The higher AIP level (5th quintile vs. 1st quintile—in men and 4th quintile vs. 1st quintile—in women) was significantly associated with increased mortality from CVD risk in the men group (HR = 1.40) and increased all-cause mortality risk in the women group (HR = 1.36). Thus, the calculation of VAI and AIP levels is simple and universally available, which makes study results easily applicable to clinical practice.

## Data availability statement

The raw data supporting the conclusions of this article will be made available by the authors, without undue reservation.

## Ethics statement

The study was approved by the Kaunas Regional Biomedical Research Ethics Committee, Lithuania (January 11, 2005; No. 05/09) and by the Ethics Committee at University College London, UK. The patients/participants provided their written informed consent to participate in this study.

## Author contributions

AT and DL conceived the idea, collected, analyzed the data, and co-wrote the manuscript. RR, DS, and DK-B contributed to writing the manuscript and the interpretation of data. MB contributed to the study concept and design and as well as supervised the research group. All authors contributed to the article and approved the final version of it.
